# Exercise reduces hyperlipidemia-induced cardiac damage in apolipoprotein E-deficient mice via its effects against inflammation and oxidative stress

**DOI:** 10.1038/s41598-023-36145-w

**Published:** 2023-06-05

**Authors:** Zuowei Pei, Jun Ji, Yanyan Gao, Heshuang Wang, Yuanyuan Wu, Jin Yang, Qin Yang, Li Zhang

**Affiliations:** 1 Department of Central Laboratory, Central Hospital of Dalian University of Technology, No. 826 Xinan Road, Dalian, 116033 China; 2 Department of Cardiology, Central Hospital of Dalian University of Technology, Dalian, 116033 China; 3 Department of Nephrology, Zhong Da Hospital, Southeast University School of Medicine, Nanjing, 210009 Jiangsu China

**Keywords:** Molecular biology, Cardiology, Endocrinology

## Abstract

Cardiovascular disease is a high incidence and mortality rate disease worldwide. Exercise training has become an established evidence-based treatment strategy that is beneficial for many cardiovascular diseases. This study aimed to investigate the effects of exercise on hyperlipidemia-induced cardiac damage in apolipoprotein E-deficient (ApoE^−/−^) mice. Male ApoE^−/−^ mice were randomly divided into the following four groups: normal diet (ND), normal diet + exercise training (ND + E), high-fat diet (HFD), and high-fat diet + exercise training (HFD + E). Exercise training consisted of swimming for 40 min, 5 days/week for 12 weeks. After 12 weeks, histopathological alterations in cardiac tissue and the serum were measured. Furthermore, the NOX4, NRF2, SIRT1, TGF-β, HO-1, collagen III, Smad3, Bax, Bak, Bcl-2, Bcl-xl, IL-1β, IL-6, and IL-18 expression levels were evaluated using immunohistochemistry and western blotting; Results: the serum levels of SIRT1, GSH-Px, and SOD were lower in ApoE^−/−^ HFD mice compared with those in ApoE^−/−^ HFD + E mice. Significant pathological changes were observed in the ApoE^−/−^ HFD + E group compared with those in the ApoE^−/−^ HFD group. Increased levels of oxidative stress, fibrosis, and apoptosis, and decreased antioxidant expression in the ApoE^−/−^ HFD group compared with those in ApoE^−/−^ HFD + E mice. Exercise exerts protective effects against cardiac damage caused by hyperlipidemia.

## Introduction

Cardiovascular disease is a common disease with a high incidence and death rate, worldwide^1^. According to the World Health Organization, nearly 23.6 million people will die of cardiovascular disease per year by 2030^2^. Dyslipidemia is a well-established risk factor for cardiovascular diseases^3^. Considerable evidence shows that hyperlipidemia leads to a chronic state of systemic inflammation, which increases the risk of myocardial inflammation, further exacerbating hyperlipidemia-associated cardiovascular diseases^4,5^. Hyperlipidemia is an abnormality in lipid metabolism, characterized by an increase in the levels of total cholesterol (TC), triglycerides (TG), and low-density lipoprotein cholesterol (LDL-c) and/or a decrease in the circulating levels of high-density lipoprotein cholesterol ^6^. Management of hyperlipidemia includes exercise, diet control, and pharmaceutical therapies, such as statin therapies^7,8^.

In the past few decades, exercise training has developed into an established evidence-based treatment strategy that is beneficial to the prognosis of many cardiovascular diseases. Thompson et al. reported the beneficial effects of increased physical activity and exercise on cardiovascular morbidity and mortality^9^. A meta-analysis reported that endurance and resistance training lowered blood pressure^10^. A review article concluded that aerobic resistance exercise and the combination of aerobic and resistance training affect cholesterol and blood lipid levels^11^. In addition, the effects of exercise have been reported in animal disease models. Korte et al. reported that exercise improves ventricular function in pigs with diabetic dyslipidemia^12^. Exercise has also been shown to reduce the development of atherosclerotic lesions in mice^13^. However, if exercise has beneficial effects on hyperlipidemia-induced cardiac damage in ApoE^−/−^ mice and the specific underlying mechanisms have not been investigated.

Apolipoprotein E-deficient (ApoE^−/−^) mice have been used as models of atherosclerosis^14^. Therefore, we established a hyperlipidemia-induced cardiac damage model using ApoE^−/−^ mice to determine the effect of exercise on hyperlipidemia-induced cardiac damage and the specific underlying mechanism.

## Results

### Exercise ameliorated cardiac dysfunction in hyperlipidemia-induced cardiac damage

**Figure 1:**
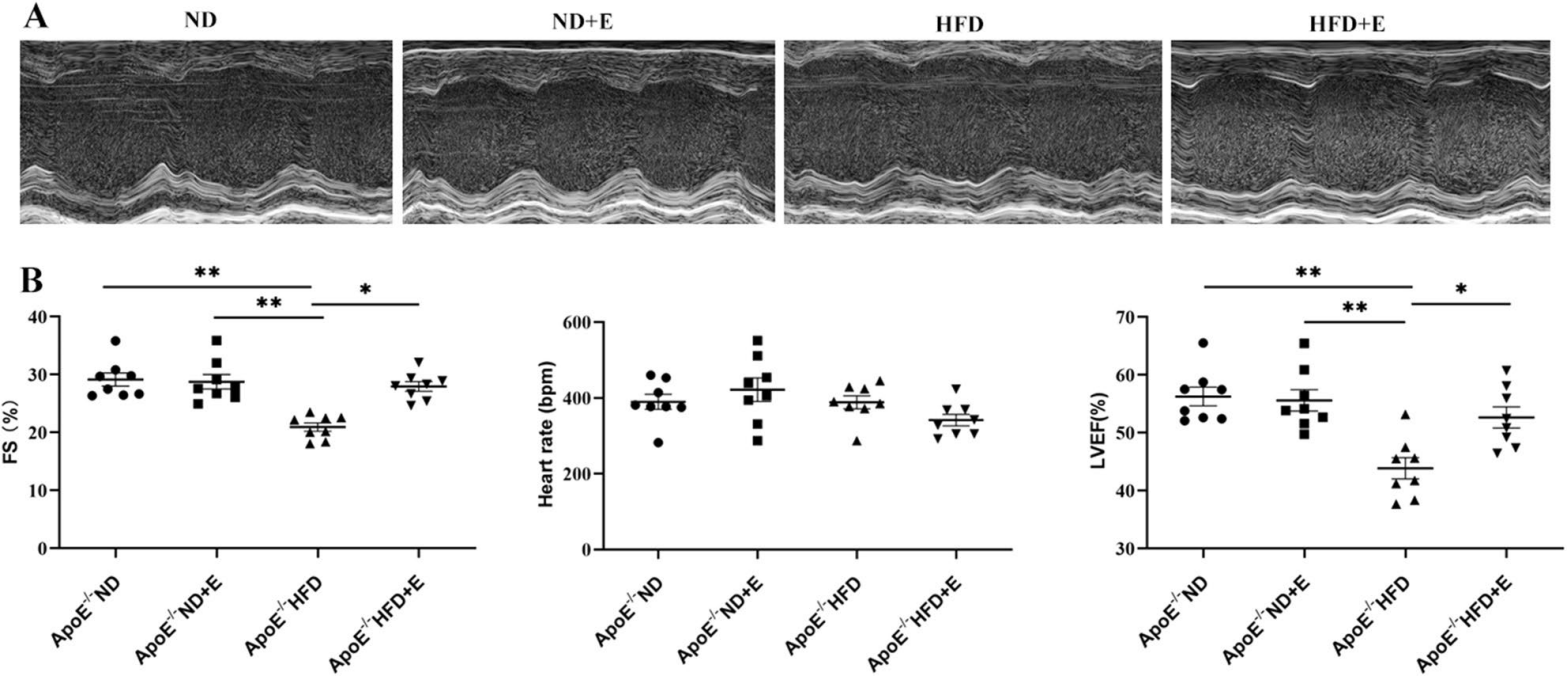
Cardiac left ventricular dysfunction in cardiac tissue after different treatments in the four mice groups. (**A**) Representative left ventricular m-mode echocardiography indicating morphological damage in the cardiac tissues of mice with different treatments. (**B**) Bar graph showing the FS%, heart rate, and LVEF% in the different mice groups. Data are shown as the mean ± SEM; n = 8 per group. *P < 0.05; **P < 0.01. *FS* fraction shortening, *LVEF* left ventricular ejection fraction, *ApoE* apolipoprotein E, *HFD* high-fat diet, *ND* normal diet, *E* exercise training.

Left ventricular m-mode echocardiography was performed to evaluate left ventricular dysfunction in cardiac tissue (Fig. 1). The left ventricular fractional shortening (FS) and left ventricular ejection fraction (LVEF) of the HFD + E group were significantly higher than those of the HFD group, indicating that exercise ameliorated cardiac dysfunction in the HFD + E group.

### Metabolic characterization

**Figure 2:**
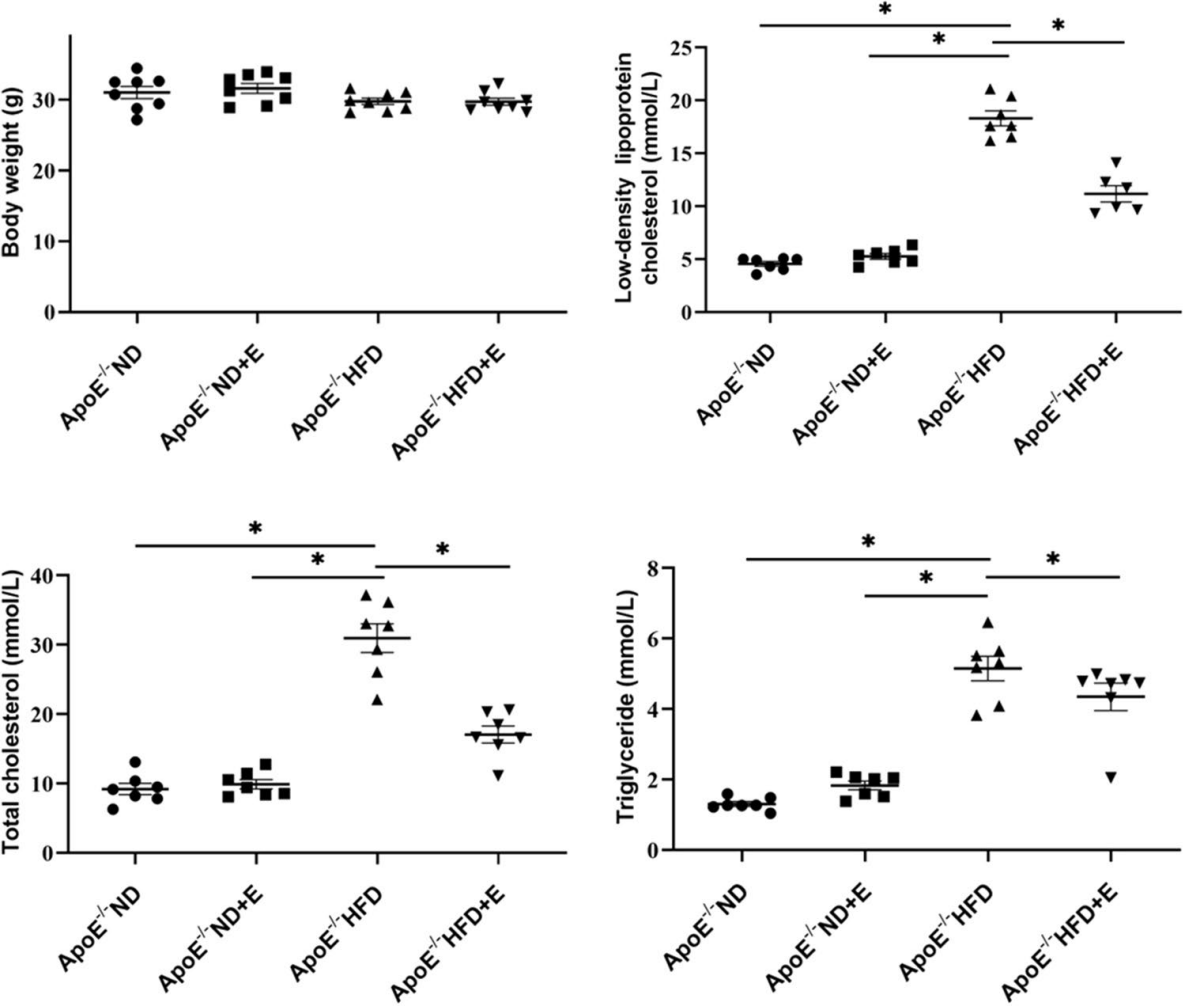
Metabolic data showing the body weights and serum TC, TG, and LDL-c levels of the mice in the four groups after different treatments. Data are shown as the mean ± SEM; n = 6–8 per Group. *P < 0.05. *TC* total cholesterol, *TG* triglyceride, *LDL-c* low-density lipoprotein cholesterol, *ApoE* apolipoprotein E, *HFD* high-fat diet, *ND* normal diet, *E* exercise training.

The metabolic characteristics of the mice in the four groups are shown in Fig. 2. Body weight did not differ among the four groups. A significant increase in the serum TG, LDL-c, and TC levels was observed in the ApoE^−/−^ HFD group compared with those in the ApoE^−/−^ ND group. However, exercise treatment significantly decreased the serum TG levels, LDL-c and TC levels.

### Exercise increased SIRT1 expression in cardiac tissue with hyperlipidemia-induced damage

**Figure 3:**
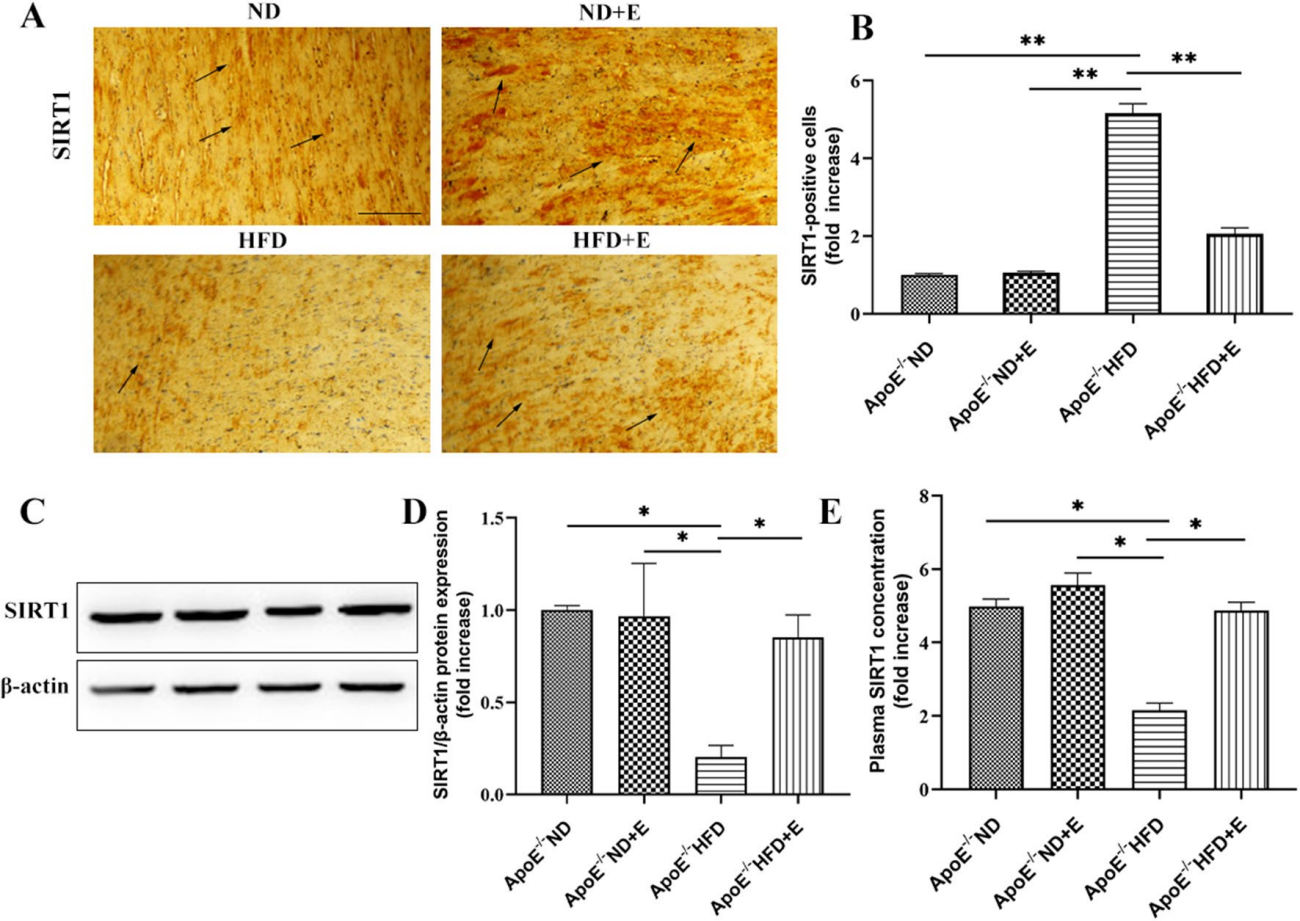
SIRT1 expression in cardiac tissue in the three groups after 12 weeks of treatment. (**A**) Immunohistochemistry for SIRT1 in cardiac tissue. Scale bar = 100 μm. Arrows indicate positively stained cells. (**B**) Bar graph showing SIRT1 positive cells. (**C**) Western blotting for SIRT1 in cardiac tissues. (**D**) Quantification of SIRT1 protein expression. (**E**) Plasma SIRT1 expression in cardiac tissues. Data are shown as the mean ± SEM; n = 7 per group, *P < 0.05; **P < 0.01. *ApoE* apolipoprotein E, *HFD* high-fat diet, *ND* normal diet, *E* exercise training, *SIRT1* sirtuin 1.

Expression of SIRT1was determined using IHC (Fig. 3A,B), western blotting (Fig. 3C,D), and ELISA kits (Fig. 3E). The SIRTl original gels are presented in Supplementary Fig. S3. Compared with ApoE^−/−^ HFD mice, ApoE^−/−^ HFD + E mice exhibited significantly increased SIRT1 expression levels, indicating that exercise increased SIRT1 expression in ApoE^−/−^ HFD mice.

### Exercise improved the histopathology of cardiac tissue with hyperlipidemia-induced damage

**Figure 4:**
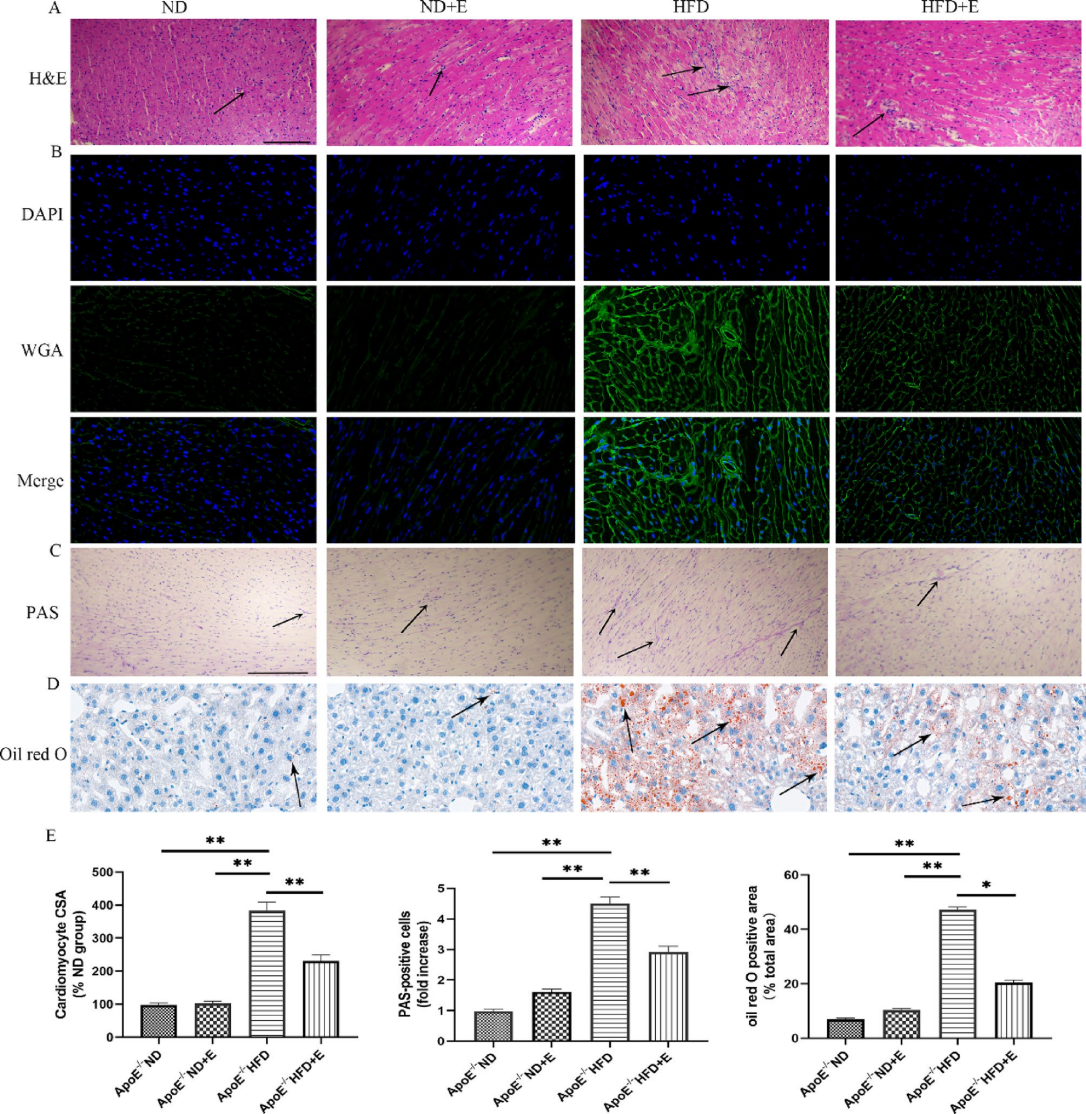
Effect of exercise on hyperlipidemia-induced cardiac damage shown using H&E, PAS, WGA, and Oil Red O staining. (**A**) Exercise attenuated inflammatory cell infiltration in HFD + E group mice compared with that in ApoE^−/−^ HFD group mice. Scale bar = 100 μm. Arrows indicate positively stained cells. (**B**) WGA-stained (green fluorescence) and DAPI-stained (blue fluorescence) cardiac tissue sections obtained at × 40 magnification. (**C**) PAS staining in cardiac tissues. Scale bar = 100 μm. Arrows indicate positively stained cells. (**D**) Oil Red O staining of cardiac tissue sections obtained at × 40 magnifications. (**E**) Bar graph showing differences in the CSA of cardiomyocytes and percentage of PAS and Oil Red O positive cells, among different groups. Data are shown as the mean ± SEM; n = 3 per group, *P < 0.05; **P < 0.01. *ApoE* apolipoprotein E, *HFD* high-fat diet, *ND* normal diet, *E* exercise training, *H&E* hematoxylin and eosin, *PAS* periodic acid-Schiff, *WGA* wheat germ agglutinin, *CSA* cross-sectional area.

H&E, WGA, PAS, and Oil Red O staining were used to evaluate histopathological changes in cardiac tissues (Fig. 4). H&E, PAS, and Oil Red O staining showed that cardiac tissue samples from ApoE^−/−^ND mice were normal. However, pathological lesions were observed in the cardiac tissue samples of ApoE^−/−^HFD mice, which included heart lipid deposition and pro-inflammatory cell infiltration. Notably, this damage was suppressed in ApoE^−/−^ HFD + E mice. In addition, the results of WGA staining showed that the cardiomyocytes were not orderly arranged, and that the cross-sectional areas (CSAs) of cardiomyocytes were significantly enlarged in ApoE^−/−^ HFD mice, while exercise could prevent these pathomorphological changes.

### Exercise inhibited oxidative stress in cardiac tissues with hyperlipidemia-induced damage

**Figure 5:**
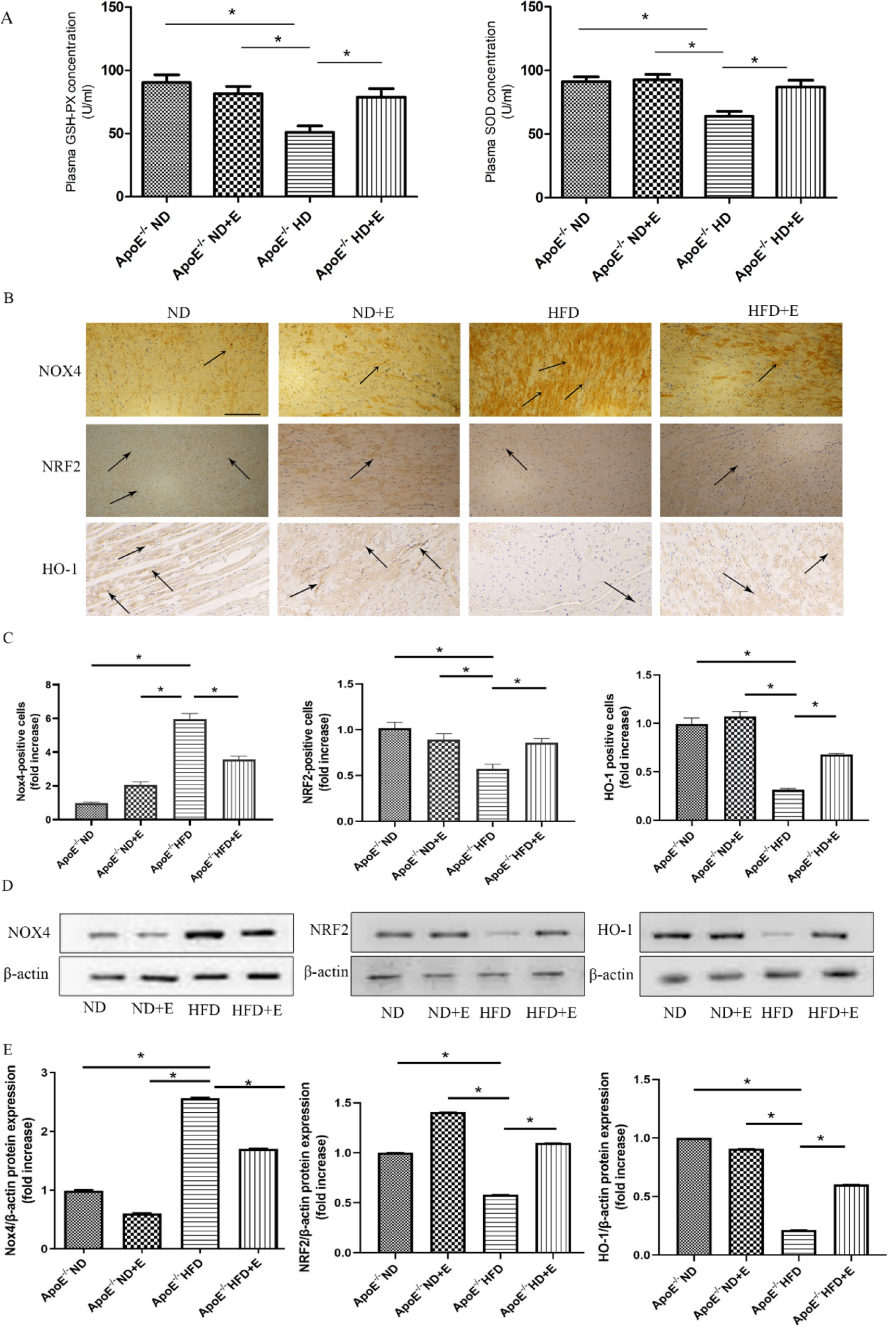
Effect of exercise on hyperlipidemia-induced cardiac oxidative stress. (**A**): GSH-Px and SOD levels in the four mouse groups after 12 weeks of different treatments. (**B**): Representative immunohistochemistry staining for NOX4, NRF2, and HO-1 in cardiac tissue of mice with different treatments. Scale bar = 100 μm. Arrows indicate positively stained cells. (**C**): NOX4, NRF2, and HO-1 positive cells. Data represent the mean ± SEM; n = 7 per group. (**D**) Western blotting for NOX4, NRF2. and HO-1 protein expression in cardiac tissue. (**E**) Quantification of NOX4, NRF2, and HO-1 protein expression. Data represent the mean ± SEM; n = 3 per group. *P < 0.05. *GSH-Px* glutathione peroxidase, *SOD* superoxide dismutase, *HO-1* heme oxygenase 1, *NRF2* nuclear factor erythroid 2-related factor, *NOX4* NADPH Oxidase 4.

Oxidative stress-related indicators were evaluated in cardiac tissues. The plasma GSH-Px and SOD levels of the subjects are shown in Fig. 5A. The ApoE^−/−^ HFD group showed markedly decreased GSH-Px and SOD levels compared with those of the ApoE^−/−^ ND and ApoE^−/−^ ND + E groups. However, the GSH-Px and SOD levels significantly increased after exercise treatment. The levels of NOX4, NRF2, and HO-1 in cardiac tissues were determined using IHC and western blotting (Fig. 5B–E). The NOX4, NRF2, and HO-1 original gels are presented in Supplementary Fig. S5. Our results showed that the levels of NOX4 in ApoE^−/−^ HFD + E group mice were significantly suppressed compared with those in ApoE^−/−^ HFD group mice. However, the opposite trend was observed in the levels of NRF2 and HO-1; the expression levels of NRF2 and HO-1 were decreased in ApoE^−/−^ HFD + E group mice compared with those in ApoE^−/−^ HFD group mice. This result indicated that exercise reduced NOX4 expression and increased NRF2 and HO-1 expression in ApoE^−/−^ HFD mice.

### Exercise inhibited collagen deposition and fibrosis in cardiac tissue with hyperlipidemia-induced damage

**Figure 6:**
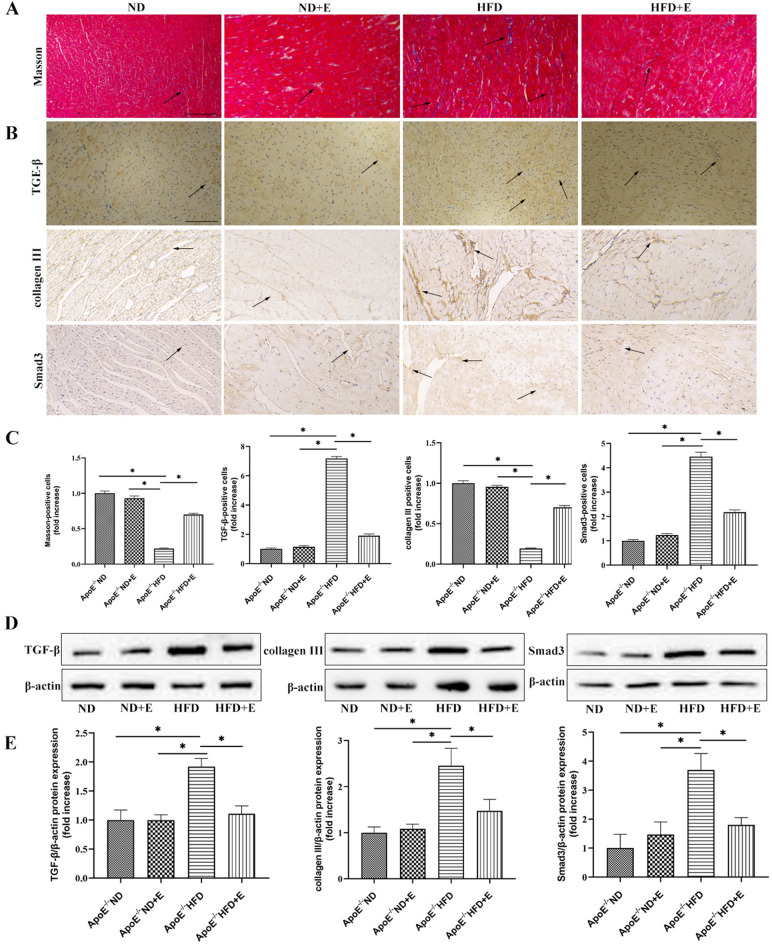
Effect of exercise on hyperlipidemia-induced cardiac fibrosis. (**A**) Fibrosis was evaluated using Masson’s trichrome staining. Scale bar = 100 μm. Arrows indicate positively stained cells. (**B**) Representative immunohistochemistry staining for TGF-β, collagen III, and Smad3. Scale bar = 100 μm. Arrows indicate positively stained cells. (**C**) Bar graph showing Masson’s trichrome, TGF-β, collagen III and Smad3 positive cells. (**D**) Western blotting for TGF-β, collagen III, and Smad3 protein expression in cardiac tissue. (**E**) Quantification of TGF-β, collagen III, and Smad3 protein expression. Data represent the mean ± SEM; n = 3 per group. *P < 0.05. *ND* normal diet, *E* exercise training, *HFD* high-fat diet, *ApoE* apolipoprotein E, *TGF-β* transforming growth factor-β.

To investigate the mechanism of fibrosis in cardiac damage, Masson’s trichrome staining (Fig. 6A,C), immunohistochemical staining (Fig. 6B,C), and western blotting (Fig. 6D,E) for TGF-β, collagen III, and Smad3 were performed.The TGF-β, collagen III, and Smad3 original gels are presented in Supplementary Fig. S6.  Masson’s trichrome staining showed that collagen deposition increased significantly in cardiac tissues, suggesting that hyperlipidemia induced fibrosis. Compared with ApoE^−/−^ HFD mice, ApoE^−/−^ HFD + E mice exhibited significantly reduced TGF-β, collagen III, and Smad3 expression levels, indicating that exercise reduced TGF-β, collagen III, and Smad3 expression in ApoE^−/−^ HFD mice. Taken together, these data suggest that exercise attenuated fibrosis in cardiac tissues with hyperlipidemia-induced damage.

### Exercise inhibited inflammation in cardiac tissues with hyperlipidemia-induced damage

**Figure 7:**
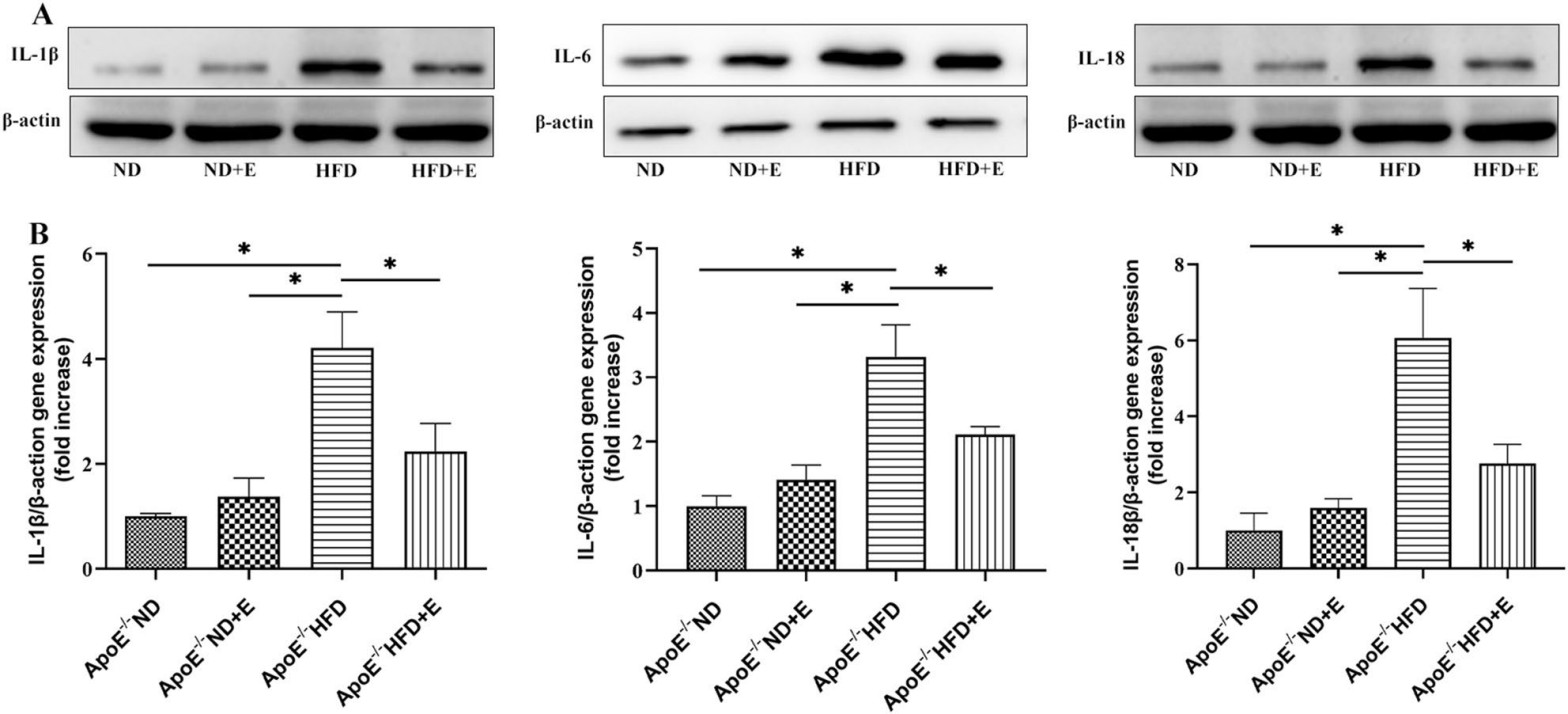
Effect of exercise on hyperlipidemia-induced cardiac inflammation. (**A**) Western blotting for IL-1β, IL-6, and IL-18 protein expression in cardiac tissue. (**B**) Bar graph showing quantification of IL-1β, IL-6, and IL-18 protein expression. The  IL-1β, IL-6, and IL-18 original gels  are presented in Supplementary Fig. S7. Data represent the means ± SEM; n = 3 per group. *P < 0.05. *ND* normal diet, *E* exercise training, *HFD* high-fat diet, *ApoE* apolipoprotein E, *IL* interleukin.

To investigate the mechanism of inflammation in cardiac damage, western blotting for IL-1β, IL-6, and IL-18 (Fig. 7A,B) was performed. Compared with ApoE^−/−^ HFD mice, ApoE^−/−^ HFD + E mice exhibited significantly reduced IL-1β, IL-6, and IL-18 expression levels, indicating that exercise reduced inflammation in ApoE^−/−^ HFD mice.

### Exercise inhibited apoptosis in cardiac tissues with hyperlipidemia-induced damage

**Figure 8:**
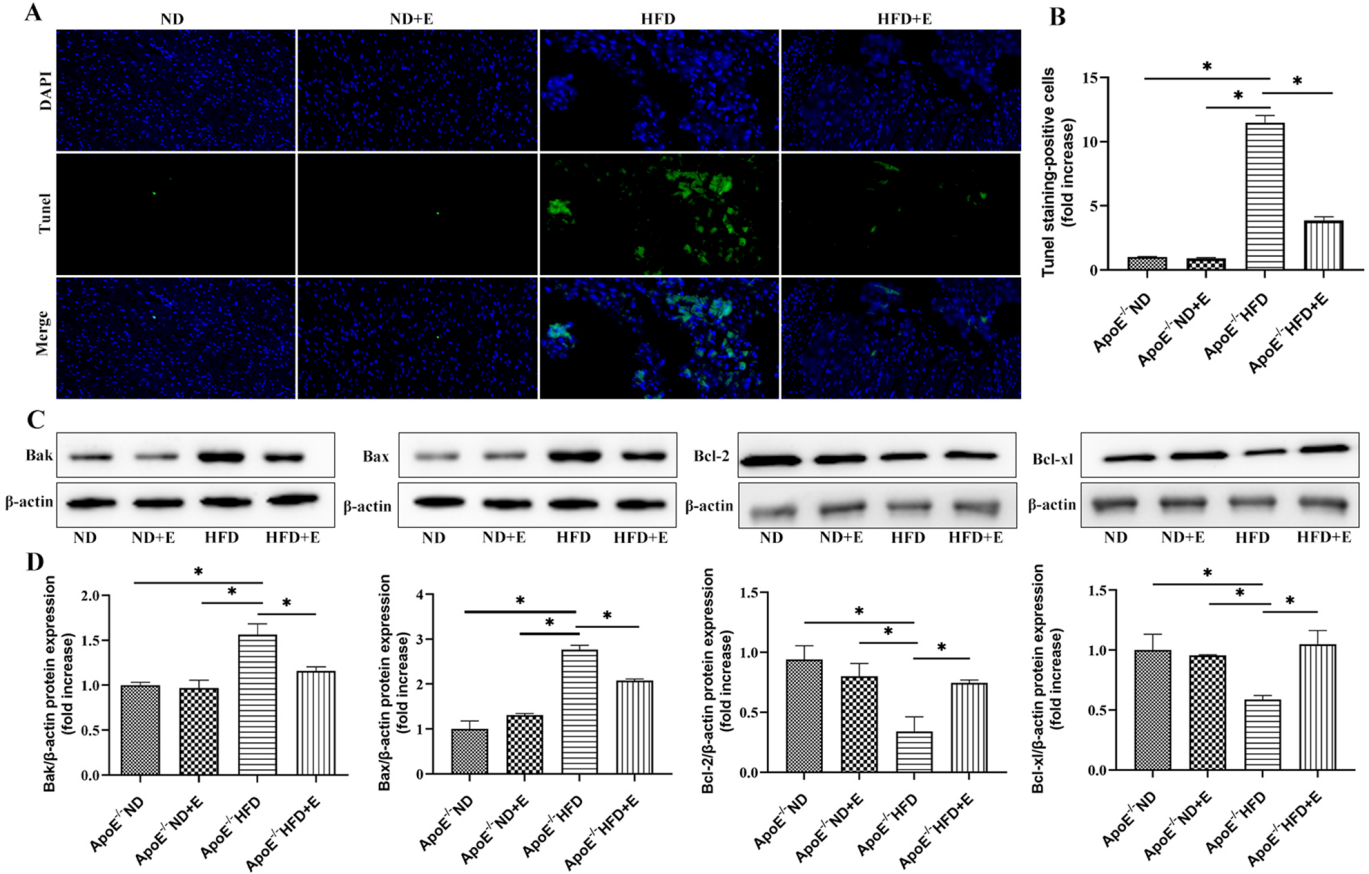
Effect of exercise on hyperlipidemia-induced cardiac apoptosis. (**A**) Apoptosis was examined using TUNEL staining. Green, TUNEL-positive nuclei; blue, nuclei. Scale bar = 100 μm. (**B**) Quantitative analysis of cell death. (**C**) Western blotting for Bak, Bax, Bcl-2, and Bcl-xl protein expression in cardiac tissue. (**D**) Bar graph showing quantification of Bak, Bax, Bcl-2, and Bcl-xl protein expression. Data represent the mean ± SEM; n = 3 per group. *P < 0.05. *ND* normal diet, *E* exercise training, *HFD* high-fat diet, *ApoE* apolipoprotein E.

TUNEL staining (Fig. 8A,B), performed to investigate the mechanism of apoptosis in cardiac damage, showed that the number of TUNEL-positive cells was higher in ApoE^−/−^ HFD mice than in ApoE^−/−^ ND mice. However, apoptosis was suppressed by exercise. Western blotting (Fig. 8C,D) were performed to evaluate Bak, Bax, Bcl-2 and Bcl-xl expression in cardiac tissues. The Bak, Bax, Bcl-2, and Bcl-xl original gels are presented in Supplementary Fig. S8. Compared with ApoE^−/−^ ND mice, ApoE^−/−^ HFD mice exhibited significantly increased Bak and Bax expression levels, which were downregulated by exercise in ApoE^−/−^ HFD + E group mice. However, the levels of Bcl-2 and Bcl-xl showed the opposite trend. Increased Bcl-2 and Bcl-xl levels were observed in ApoE^−/−^ HFD + E group mice compared with those in ApoE^−/−^ HFD group mice. Taken together, these data suggest that exercise attenuated apoptosis in hyperlipidemia-induced cardiac tissues.

## Discussion

**Figure 9:**
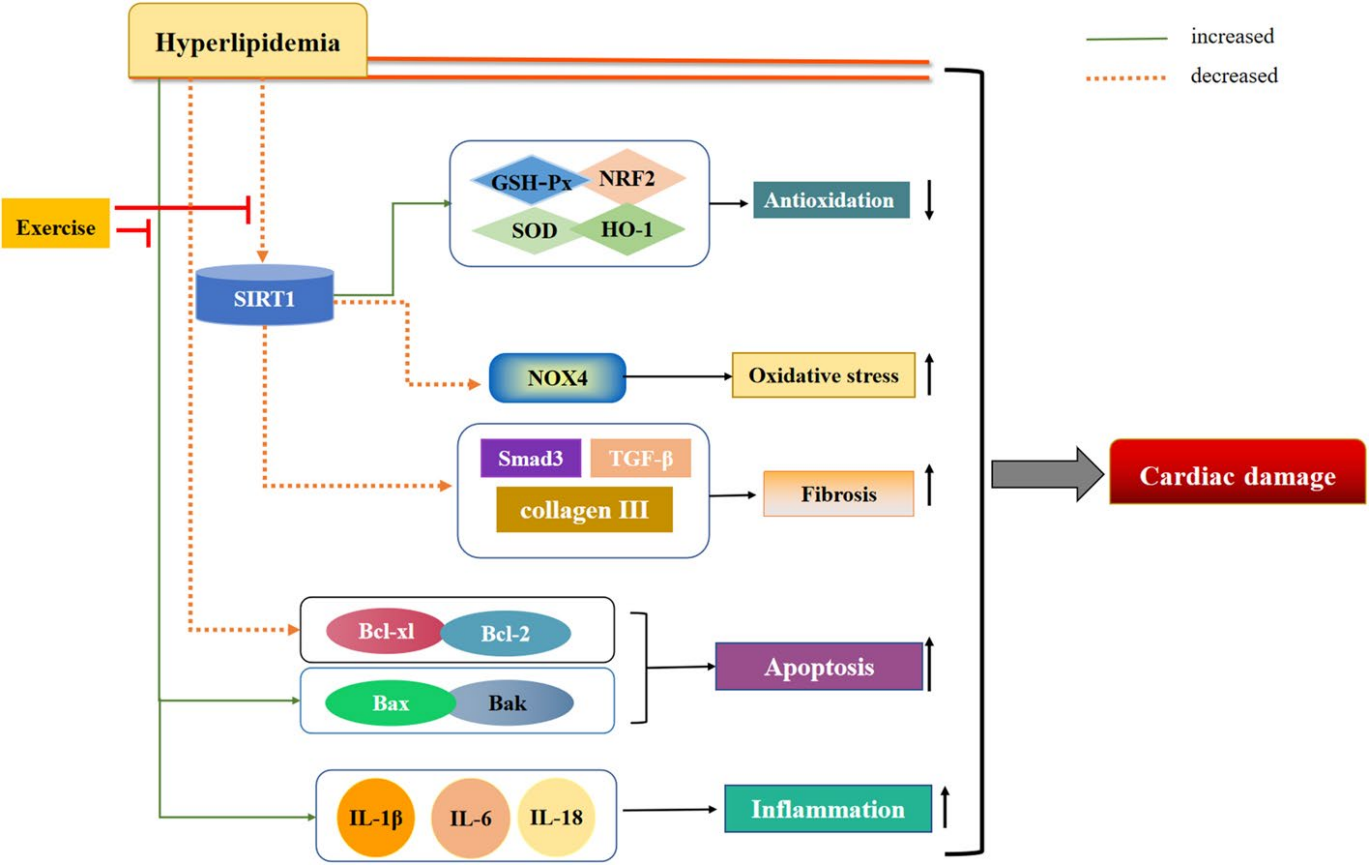
Schematic representation of the mechanism by which exercise protects against cardiac damage induced by hyperlipidemia. *SIRT1* sirtuin 1, *GSH-Px* glutathione peroxidase, *SOD* superoxide dismutase, *HO-1* heme oxygenase 1, *NRF2* nuclear factor erythroid 2-related factor, *NOX4* NADPH Oxidase 4, *TGF-β* transforming growth factor-β, *IL* interleukin.

This study provides the evidence that exercise could attenuate hyperlipidemia-induced leukocyte infiltration, collagen accumulation, lipid deposition, oxidative stress, fibrosis, and apoptosis, as well as reduce the downregulation of SIRT1, SOD, and GSH-Px (Fig. 9). In the present study, the protective effects of exercise against hyperlipidemia-induced cardiac damage were explored in a mouse model of hyperlipidemia-induced cardiac damage. Compared with ApoE^−/−^ ND + E group mice, LDL-c and TC levels were significantly lower in ApoE^−/−^ HFD group mice, suggesting that exercise has a protective effect against cardiac damage by progressive lipid deposition. H&E, Masson’s trichrome, PAS, and Oil Red O staining results showed increased leukocyte infiltration, collagen accumulation, and lipid deposition in ApoE^−/−^ HFD group mice compared with those in ApoE^−/−^ HFD + E group mice. Based on metabolic characterization and histopathological changes, it was observed that cardiac damage occurs with a HFD, but exercise attenuates this damage.

Cardiovascular disease is a widespread disease with a high incidence and death rate worldwide^1^. Dyslipidemia is a well-established risk factor for cardiovascular disease that may further exacerbate cardiovascular disease^3^. Overexpression of endothelial sirtuin 1 (SIRT1) may prevent atherosclerosis by improving vascular function^15^. Stein et al. found that SIRT1 deletion in bone marrow-derived macrophages may promote atherogenesis^16^. These results are consistent with those of our study. ApoE^−/−^ HFD mice showed a significant decrease in the SIRT1 levels compared with those in ApoE^−/−^ ND mice. However, this decrease was attenuated in ApoE^−/−^ HFD + E group mice. Taken together, these results demonstrate that exercise can reduce hyperlipidemia-induced cardiac damage by increasing the expression of SIRT1.

Previous studies pointed out that hypertriglyceridemia is accompanied by the destruction of antioxidant enzyme defense, which may lead to cell and tissue damage^17^. In addition, evidence suggests that oxidative stress plays a significant role in doxorubicin-induced myocardial injury^18^. Furthermore, to analyze the effects of oxidative stress on hyperlipidemia-induced cardiac damage and investigate the protective effects of exercise on cardiac damage, serum SOD and GSH-Px levels were determined using an ELISA kit. The defense system of SOD and GSH-Px antioxidant enzymes is the first line of defense against oxidative injury in mammalian systems^19,20^. In this study, exercise was found to increase the SOD and GSH-Px levels, which is consistent with the findings of Lu et al.^21^.

SIRT1 regulates key cellular processes, including antioxidant defense, DNA repair, and genomic stability^22^. Oxidative stress is characterized by an imbalance between reactive oxygen species (ROS) production and oxidative stress resistance^23^. The main form of ROS is the superoxide anion (O_2_^−^), and the NADPH oxidase (NOX) family is directly related to the generation of ROS^24,25^. Kuroda et al. indicated that the NOX4-ROS pathway could aggravate remodeling processes and worsen cardiac function in failing hearts^26^. However, it has been confirmed that inhibition of SIRT1 causes upregulation of p22phox and NOX4, eventually leading to O_2_ production^27,28^. Xu et al. reported that salidroside inhibits psoriasis-associated oxidative stress by activating SIRT1^29^. Therefore, we detected the expression of NOX4 using IHC and western blotting. The results showed that exercise suppressed the increase in NOX4 levels in the ApoE^−/−^ HFD group. In addition, the increase in oxidative stress is regulated by cellular defense mechanisms that are controlled by the NRF2-Keap1 signaling pathway. Increased cell ROS levels cause the Keap1 protein to dissociate from nuclear factor erythroid 2-related factor (NRF2), causing NRF2 to translocate to the nucleus^30^. NRF2 has been shown to be an emerging regulator of the cellular expression of a number of genes encoding antioxidant enzymes, anti-apoptotic proteins, and drug transporters that regulate the expression of a variety of cellular protective proteins, such as antioxidant enzymes, including heme oxygenase 1 (HO-1)^31,32^. Chen et al. in their review study demonstrated the cardioprotective effects of NRF2^33^. In addition, Kosuru et al. showed that pterostilbene increases the expression of NRF2 to reduce cardiac oxidative stress and inflammation in fructose-fed rats^34^. In this study, exercise was found to decrease cardiac damage by increasing the expression of NRF2 and HO-1.

Pharmacological activation of SIRT1 has been reported as a new therapeutic strategy for the prevention of cardiac fibrosis^35^. Liu et al. reported that SIRT1 activation attenuates cardiac fibrosis via its effects on endothelial-to-mesenchymal transition^36^. Cappetta et al. showed that SIRT1 may interfere with cardiac fibroblast activation by inhibiting the P-Smad3 pathway, ultimately reducing cardiac fibrosis^37^. Cardiac fibrosis is a common pathological feature of heart disease and can lead to serious cardiac dysfunction^38^. Emerging evidence has demonstrated that the transforming growth factor-β (TGF-β)/Smad signaling pathway plays a critical role in cardiac fibrosis^39^. Therefore, to investigate whether the activation of SIRT1 could attenuate hyperlipidemia-induced cardiac fibrosis in vivo and whether the TGF-β/Smad pathway is involved in this process, Masson’s trichrome staining and collagen III, TGF-β, and Smad3 expression were assessed. Masson’s trichrome staining, IHC, and western blot analysis of collagen III showed that exercise suppressed collagen accumulation in the ApoE^−/−^ HFD group. In addition, the expression of TGF-β and Smad3 was decreased in the ApoE^−/−^ HFD + E group compared with that in the ApoE^−/−^ HFD group. TGF-β is a pleiotropic cytokine that participates in various cellular functions^40^. It has been reported that excessive TGF-β may be harmful by promoting extracellular matrix deposition, increased myocardial stiffness, and diastolic dysfunction^41^. Hence, exercise increases the level of SIRT1, and the upregulation of SIRT1 reduces hyperlipidemia-induced cardiac fibrosis and collagen accumulation.

Furthermore, HE staining showed higher inflammatory cell infiltration in the HFD group compared with that in the control group. Interestingly, exercise reduced this infiltration in the ApoE^−/−^ HFD + E group. In addition, exercise reduced the hyperlipidemia-induced increase in the expression of inflammatory signals, including IL-1β, IL-6, and IL-18, in kidney tissue. Mao et al. showed that TQ reduced the levels of IL-1-β, IL-6, and IL-18 in mice with PM2.5-induced lung injury, consistent with our results^42^. Proinflammatory cytokines are major mediators of hyperlipidemia-induced cardiac damage^43^. Therefore, inhibition of proinflammatory cytokine release may be an effective approach to treat hyperlipidemia-induced cardiac damage.

Previous studies have demonstrated that lipid deposition causes oxidative stress, resulting in increased ROS production and protein and DNA damage, leading to increased apoptosis^44,45^. Furthermore, studies have shown that SIRT1 and NRF2 regulate apoptotic proteins^46^. As mentioned earlier, severe oxidative stress changes were observed in cardiac tissue; therefore, the levels of apoptosis-related proteins were further examined. TUNEL staining showed that the number of TUNEL-positive cells was lower in ApoE^−/−^ HFD + E mice than that in ApoE^−/−^ HFD mice. ApoE^−/−^ HFD + E mice exhibited significantly decreased Bak and Bax expression levels and increased Bcl-2 and Bcl-xl expression levels. These data suggest that exercise attenuates cardiac apoptosis in hyperlipidemia-induced cardiac tissues.

However, this study had some limitations. Only an in vivo study was performed and cell experiments were not included to explore the specific mechanism underlying the protective effects of exercise on hyperlipidemia-induced cardiac tissues. Therefore, we plan to perform cell experiments in a future study.

## Conclusions

In conclusion, the present study showed that exercise had a protective effect against hyperlipidemia-induced cardiac damage in ApoE^−/−^ mice. Exercise reduced the downregulation of SIRT1, SOD, and GSH-Px and protected against cardiac damage by inhibiting leukocyte infiltration, collagen accumulation, lipid deposition and inflammation. In addition, exercise increased the level of SIRT1, and then the up-regulation of SIRT1 reduced hyperlipidemia-induced cardiac oxidative stress, fibrosis, inflammation and apoptosis. The findings of this study, we performed swimming training in mice and found that the intensity of swimming could be beneficial for developing novel strategies to prevent and treat cardiac damage.

## Materials and methods

### Animals

This study was performed in accordance with the ARRIVE (Animal Research: Reporting of In Vivo Experiments) guidelines 2.0 (https://arriveguidelines.org). Eight-week-old male ApoE^−/−^ mice with the body weight of 23 ± 1.35 g were obtained from Beijing Vital River Laboratories Animal Technology Co., Ltd. (Beijing, China). Animals were housed in room under a 12-h light–dark cycle within a temperature-regulated environment supplied with water ad libitum. ApoE^−/−^ mice were randomly assigned to one of the following groups: normal diet (ND group, n = 8), normal diet + exercise training (ND + E group, n = 8), high-fat diet (HFD group, n = 8), high-fat diet + exercise training (HFD + E group, n = 8). The high-fat diet consisted of a commercially prepared mouse food (MD12017) supplemented with 1.25% (wt/wt) cholesterol, 22.5% (wt/wt) protein, 20.0% (wt/wt) cocoa fat, and 45.0% carbohydrate (Jiangsu Mediscience Ltd., Jiangsu, China). One week prior to administration of the test diets, exercise training was initiated in an experimental swimming pool (temperature 30 °C; water depth 44 cm; radius 120 cm). The progressive program initially involved swimming for 5–10 min and was gradually extended to 30 min/day. When the test diet was implemented, mice were subjected to formal swimming exercise for 40 min/day, 5 days/week for 12 weeks. All animal experiments were performed in accordance with the Guide for the Care and Use of Laboratory Animals. The study was approved by the ethical committee of the Central Hospital of Dalian University of Technology.

### Echocardiography

After 12 weeks of treatment, echocardiography was performed using the Vevo 2100LT micro-ultrasound system (FUJIFILM Visual Sonics, Inc., Toronto, ONTARIO, Canada). The mice were first anesthetized with 1.5% isoflurane, after which they were immediately placed on a 37 °C thermostat to maintain normal body temperature, and slowly adjust the position and direction of the ultrasound beam to obtain an echocardiogram of the left ventricle. We acquired M-mode images to evaluate left ventricular function parameters.

### Biochemical measurements

Blood samples were taken from the abdominal aorta of rats and serum stored at – 80 °C. The levels of TC, TG, LDL-c, glutathione peroxidase (GSH-Px), superoxide dismutase (SOD) and sirtuin 1 (SIRT1) were measured using respective ELISA kits (Nanjing Jiancheng Bioengineering Institute, Nanjing, China). All assays were conducted in accordance with the manufacturer’s instructions.

### Haematoxylin and eosin (H&E) staining

Cardiac samples were fixed in 10% buffered formalin solution and embedded in paraffin. Paraffin-embedded cardiac tissue slices were deparaffinized via immersion in xylene (3 times, 5 min each), then rehydrated in a descending alcohol series. Then, serial sections (4 µm) were stained with H&E and examined microscopically using a BX40 upright light microscope (Olympus, Tokyo, Japan).

### Masson’s trichrome, FITC-conjugated wheat-germ agglutinin (WGA) and periodic acid-Schiff staining (PAS)

Mice were anesthetized with isoflurane, and hearts were fixed by perfusion with 10% buffered formalin. Hearts were fixed overnight at room temperature, transferred into 70% ethanol, and then embedded in paraffin. Paraffin-embedded cardiac tissue slices were deparaffinised via immersion in xylene (3 times, 5 min each), then rehydrated in a descending alcohol series (100, 90, 80, and 70% alcohol, 5 min each). Histology changes was detected by staining sections with Masson’s trichrome, WGA, and PAS staining. Images were acquired microscopically using a BX40 upright lightmicroscope (Olympus, Tokyo, Japan). WGA staining was used to measure the cross-sectional area of cardiomyocytes.

### Oil red O staining

The 6-μm-thick frozen sections were stained with Oil red O stain solution for 10 min, immersed them in two cups of 60% isopropanol for differentiation in turn, 3 s and 5 s respectively and then stained in Hematoxylin for 5 min. Finally, rinsed these sections in 3 cups of pure water for 5 s, 10 s, and 30 s in turn, treated it with differentiation solution (60% alcohol as solvent) for 5 s, 2 cups of distilled water for 10 s each, and Scott Tap Bluing for 1 s, prior to observation and evaluation under a microscope. The results were with Image J software and expression the oil red O positive area as a percentage of the total area.

### TUNEL staining

The hearts were embedded in paraffin, and serially sectioned to 6 μm thickness. The sections were deparaffinized and hydrated in xylene (3 times, 5 min each) and a descending alcohol series (100, 90, 80, and 70% alcohol, 5 min each), and then incubated in proteinase K (37 °C, 22 min) and stained using the Fluorescein TUNEL Cell Apoptosis Detection kit (Servicebio Technology Co., Ltd., Wuhan, China). All images were captured using a fluorescence microscope (Nikon). The cells that were positive for TUNEL staining and aligned with DAPI staining were considered apoptotic cells and counted.

### Immunohistochemistry (IHC)

Immunohistochemical analysis was performed using the HistoneSimple stain kit (Nichirei, Tokyo, Japan), according to the manufacturer’s instructions. Paraffin-embedded sections were deparaffinised with xylene and then rehydrated in a descending series of ethanol washes. The sections were treated for 15 min with 3% H_2_O_2_ in methanol to inactivate endogenous peroxidases and then incubated at 4 °C overnight with primary antibodies to SIRT1 (rabbit anti-SIRT1 antibody, 1:300; Solarbio), NRF2 (rabbit anti-NRF2 antibody, 1:200; Proteintech), NOX4 (rabbit anti-NOX4 antibody, 1:200; Proteintech), TGF-β (rabbit anti-TGF-β antibody, 1:200; Solarbio), HO-1 (rabbit anti-HO-1 antibody, 1:100; Solarbio), collagen III (rabbit anti-collagen III antibody, 1:100; Solarbio), Smad3 (rabbit anti-Smad3 antibody, 1:100; Solarbio), Bax (rabbit anti-Bax antibody, 1:50; Solarbio), Bak (rabbit anti-Bak antibody, 1:100; Solarbio), Bcl-2 (rabbit anti-Bcl-2 antibody, 1:50; Solarbio) and Bcl-xl (rabbit anti-Bcl-xl antibody, 1:100; Solarbio). All sections were examined microscopically using a BX40 upright lightmicroscope (Olympus, Tokyo, Japan).

### Western blot analysis

Western blot analysis with the antibodies against SIRT1 (rabbit anti-SIRT1 antibody, 1:1000; Solarbio), NRF2 (rabbit anti-NRF2 antibody, 1:1000; Proteintech), NOX4 (rabbit anti-NOX4 antibody, 1:1000; Proteintech), TGF-β (rabbit anti-TGF-β antibody, 1:1000; Solarbio), HO-1 (rabbit anti-HO-1 antibody, 1:1000; Solarbio), collagen III (rabbit anti-collagen III antibody, 1:1000; Solarbio), Smad3 (rabbit anti-Smad3 antibody, 1:1000; Solarbio), Bax (rabbit anti-Bax antibody, 1:1000; Solarbio), Bak (rabbit anti-Bak antibody, 1:1000; Solarbio), Bcl-2 (rabbit anti-Bcl-2 antibody, 1:1000; Solarbio), Bcl-xl (rabbit anti-Bcl-xl antibody, 1:1000; Solarbio), IL-1β (rabbit anti-IL-1β antibody, 1:1000; Solarbio), IL-6 (rabbit anti-IL-6 antibody, 1:1000; Solarbio) and IL-18 (rabbit anti-IL-18 antibody, 1:1000; Solarbio) and β-actin (rabbit anti-β-actin antibody, 1:1000; Proteintech)were used. Proteins were extracted from cardiac tissues using radio-immunoprecipitation assay buffer (P0013B; Beyotime, Shanghai, China) according to the manufacturer’s protocol. Extracts were separated by sodium dodecyl sulphate–polyacrylamide gel electrophoresis (10–15%) and transferred to a polyvinylidene difluoride (PVDF) membrane (Millipore, Bedford, MA, USA). Membranes were blocked overnight by 5% milk blocking reagent at room temperature for 2 h. The membranes were then incubated in primary antibody diluents (P0023A; Beyotime) and lightly shaken overnight at 4℃. After three washes with TBS-T (15 min each), incubating the membranes with secondary antibody (anti-rabbit Ig-G, 1:2000; Proteintech) for 1 h at 37 °C. The β-actin was used as a control of protein load. Protein levels were expressed as protein/β-actin ratios to minimize loading differences. This analysis was carried out three times independently. The intensity of the bands was quantified using ImageJ software (NIH).

### Statistical analysis

SPSS software version 23.0 (SPSS Inc., Chicago, IL, USA) was used for statistical analysis. Inter-group variation was measured by one-way analysis of variance (ANOVA), followed by individual comparison with the Tukey’s test. Statistical significance was ascribed to a P value < 0.05. Data were expressed and presented as mean and standard error of mean (mean ± SEM) (Supplementary figures).

## Supplementary Information

Supplementary Figures.

## Data Availability

All the data in this paper are available from the corresponding authors upon request.

## Abbreviations

ApoE: Apolipoprotein E
ND: Normal diet
ND + E: Normal diet + exercise training
HFD: High-fat diet
HDF + E: High-fat diet + exercise training
TC: Total cholesterol
TG: Triglyceride
LDL-c: Low-density lipoprotein cholesterol
GSH-Px: Glutathione peroxidase
SOD: Superoxide dismutase
SIRT1: Sirtuin 1
H&E: Hematoxylin and eosin
PAS: Periodic acid-Schiff
WGA: Wheat germ agglutinin
CSA: Cross-sectional area
NRF2: Nuclear factor erythroid 2-related factor
NOX4: NADPH oxidase 4
TGF-β: Transforming growth factor-β
IL: Interleukin
HO-1: Heme oxygenase-1
