# Correction: Exercise reduces hyperlipidemia-induced cardiac damage in apolipoprotein E-deficient mice via its effects against inflammation and oxidative stress

**DOI:** 10.1038/s41598-025-06634-1

**Published:** 2025-07-03

**Authors:** Zuowei Pei, Jun Ji, Yanyan Gao, Heshuang Wang, Yuanyuan Wu, Jin Yang, Qin Yang, Li Zhang

**Affiliations:** 1 Department of Central Laboratory, Central Hospital of Dalian University of Technology, No. 826 Xinan Road, Dalian, 116033 China; 2 Department of Cardiology, Central Hospital of Dalian University of Technology, Dalian, 116033 China; 3 Department of Nephrology, Zhong Da Hospital, Southeast University School of Medicine, Nanjing, 210009 Jiangsu China

**Keywords:** Molecular biology, Cardiology, Endocrinology

Correction to: *Scientific Reports* 10.1038/s41598-023-36145-w, published online 05 June 2023

The original version of this Article contained an error in Figure 4A, where due to an error in the process of naming the image during the experiments, the H&E image of ‘HFD’ group was an incorrect version of the experimental dataset. The original Figure 4 and accompanying legend appear below.

**Fig. 4:**
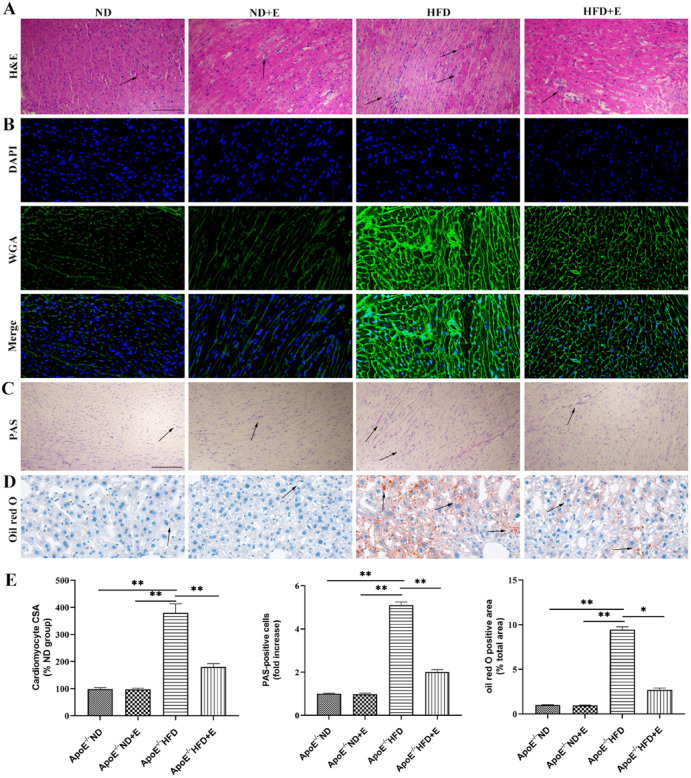
Effect of exercise on hyperlipidemia-induced cardiac damage shown using H&E, PAS, WGA, and Oil Red O staining. (**A**) Exercise attenuated inflammatory cell infiltration in HFD + E group mice compared with that in ApoE^−/−^ HFD group mice. Scale bar = 100 μm. Arrows indicate positively stained cells. (**B**) WGA-stained (green fluorescence) and DAPI-stained (blue fluorescence) cardiac tissue sections obtained at × 40 magnification. (**C**) PAS staining in cardiac tissues. Scale bar = 100 μm. Arrows indicate positively stained cells. (**D**) Oil Red O staining of cardiac tissue sections obtained at × 40 magnifications. (**E**) Bar graph showing differences in the CSA of cardiomyocytes and percentage of PAS and Oil Red O positive cells, among different groups. Data are shown as the mean ± SEM; n = 3 per group, *P < 0.05; **P < 0.01. *ApoE* apolipoprotein E, *HFD* high-fat diet, *ND* normal diet, *E* exercise training, *H&E* hematoxylin and eosin, *PAS* periodic acid-Schiff, *WGA* wheat germ agglutinin, *CSA* cross-sectional area.

Additionally, the Article contained an error in Figure 5B, where the NRF2 image under ‘ND’ group was a duplication of the NRF2 image under ‘ND + E’ group. The original Figure 5 and accompanying legend appear below.

**Fig. 5:**
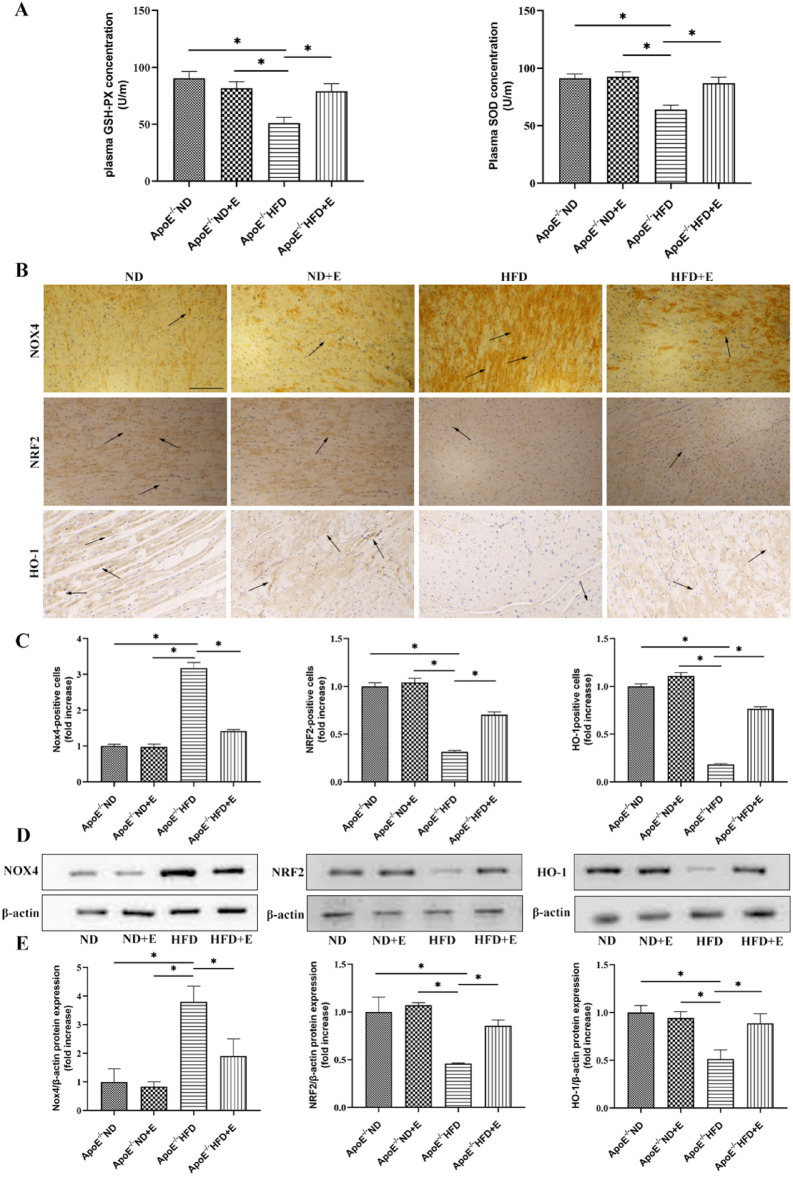
Effect of exercise on hyperlipidemia-induced cardiac oxidative stress. (**A**) GSH-Px and SOD levels in the four mouse groups after 12 weeks of different treatments. (**B**) Representative immunohistochemistry staining for NOX4, NRF2, and HO-1 in cardiac tissue of mice with different treatments. Scale bar = 100 μm. Arrows indicate positively stained cells. (**C**) NOX4, NRF2, and HO-1 positive cells. Data represent the mean ± SEM; n = 7 per group. (**D**) Western blotting for NOX4, NRF2. and HO-1 protein expression in cardiac tissue. (**E**) Quantification of NOX4, NRF2, and HO-1 protein expression. Data represent the mean ± SEM; n = 3 per group. *P < 0.05. *GSH-Px* glutathione peroxidase, *SOD* superoxide dismutase, *HO-1* heme oxygenase 1, *NRF2* nuclear factor erythroid 2-related factor, *NOX4* NADPH Oxidase 4.

The original Article has been corrected.

