# Supplementary material for: Exercise reduces hyperlipidemia-induced cardiac damage in apolipoprotein E-deficient mice via its effects against inflammation and oxidative stress
Source: Sci Rep. 2023 Jun 5;13:9134. doi: 10.1038/s41598-023-36145-w (PMC10241954; doi:10.1038/s41598-023-36145-w)
Supplement: Supplementary file 1 — Supplementary Figures. [file 41598_2023_36145_MOESM1_ESM.pdf]

Supplements

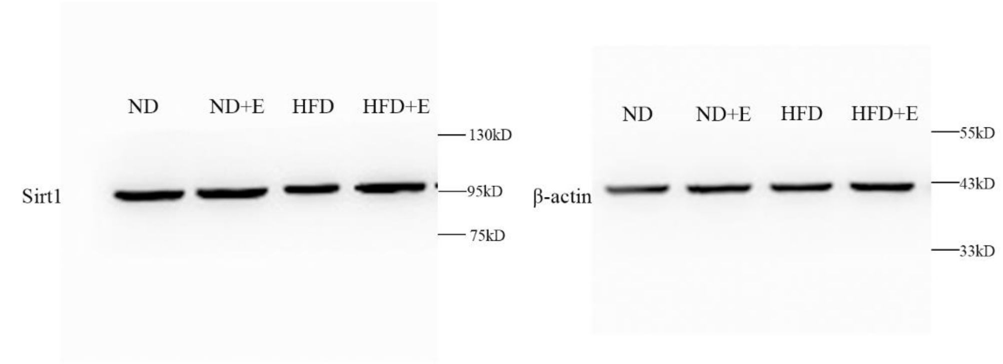

Figure S3. The SIRT1 original gels.

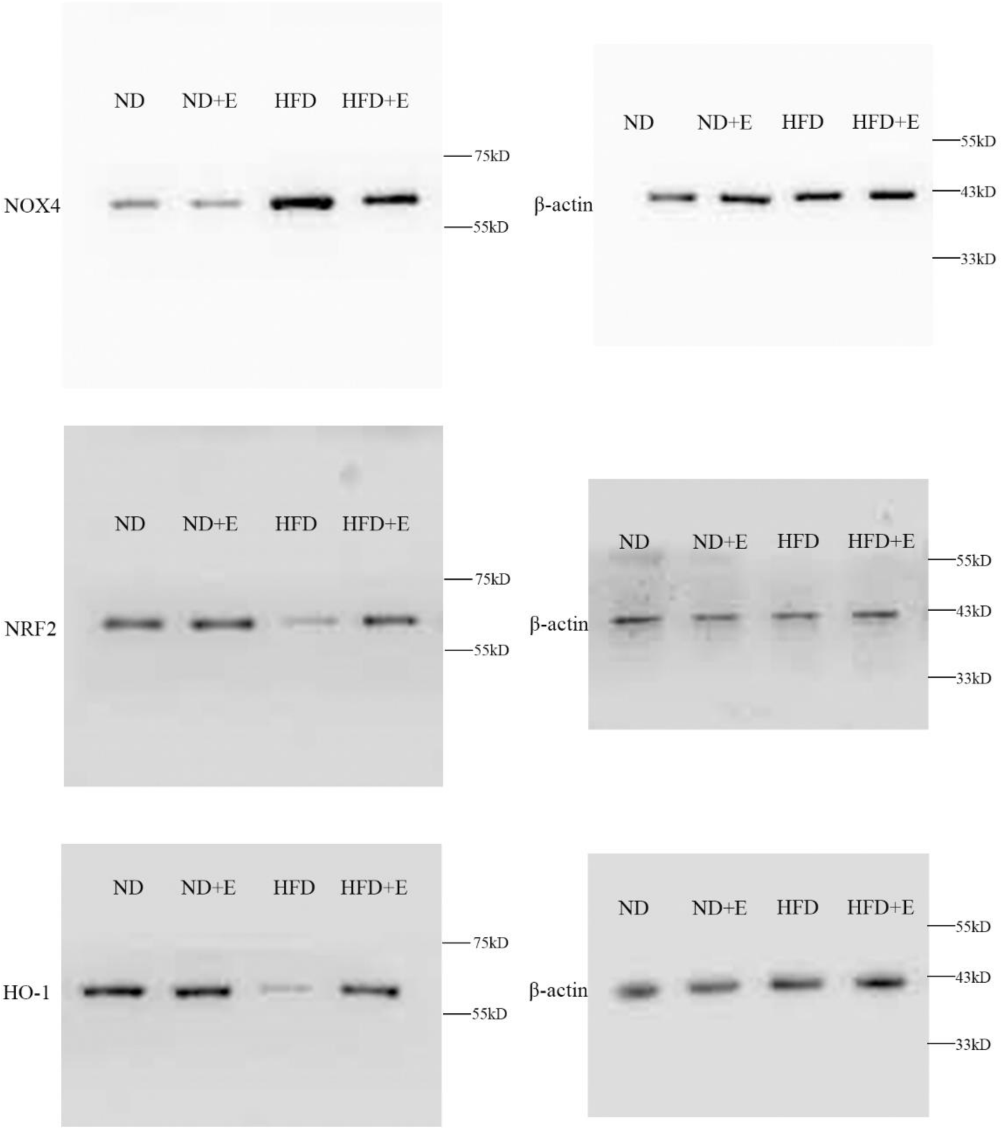

Figure S5. The NOX4, NRF2, and HO-1 original gels.

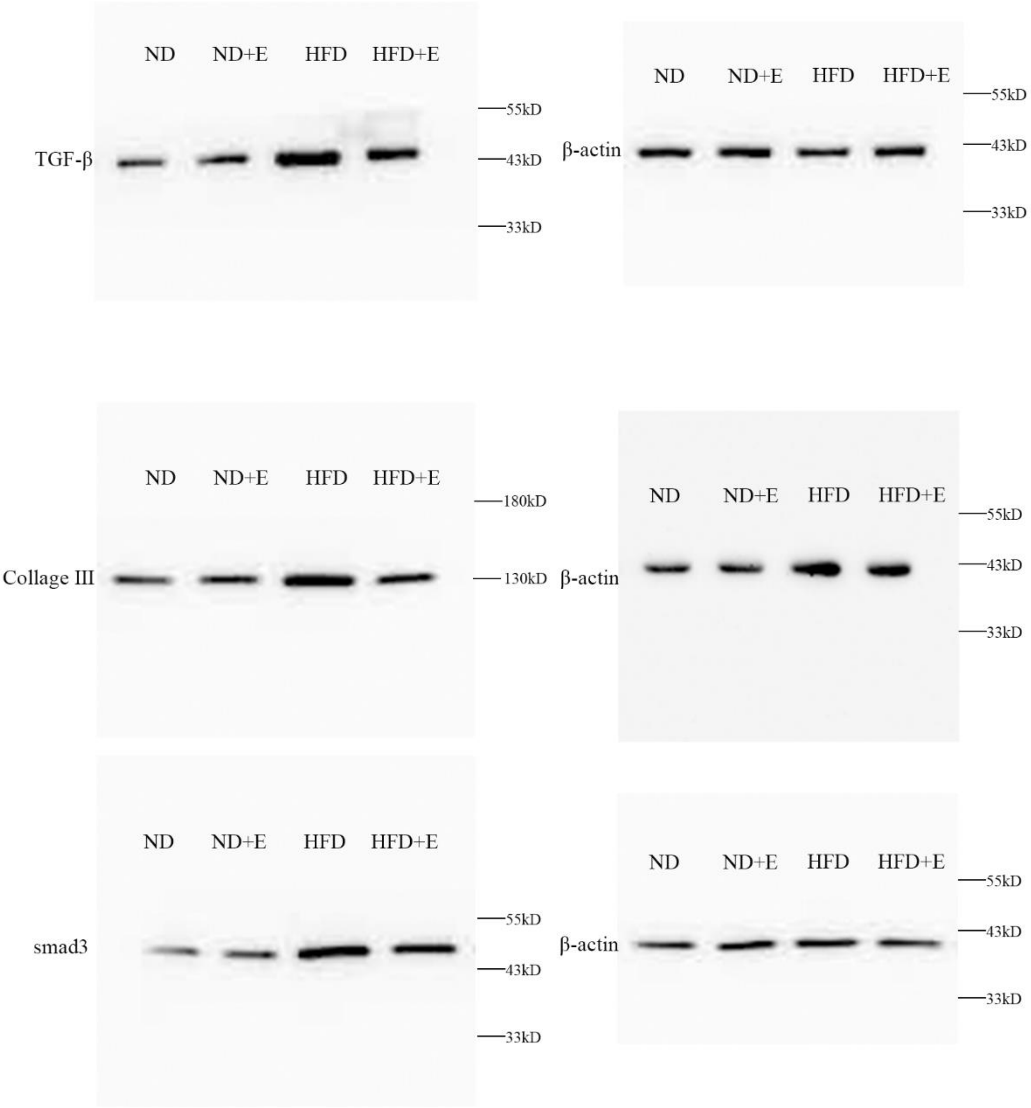

Figure S6. The TGF-β, collagen Ⅲ, and Smad3 original gels.

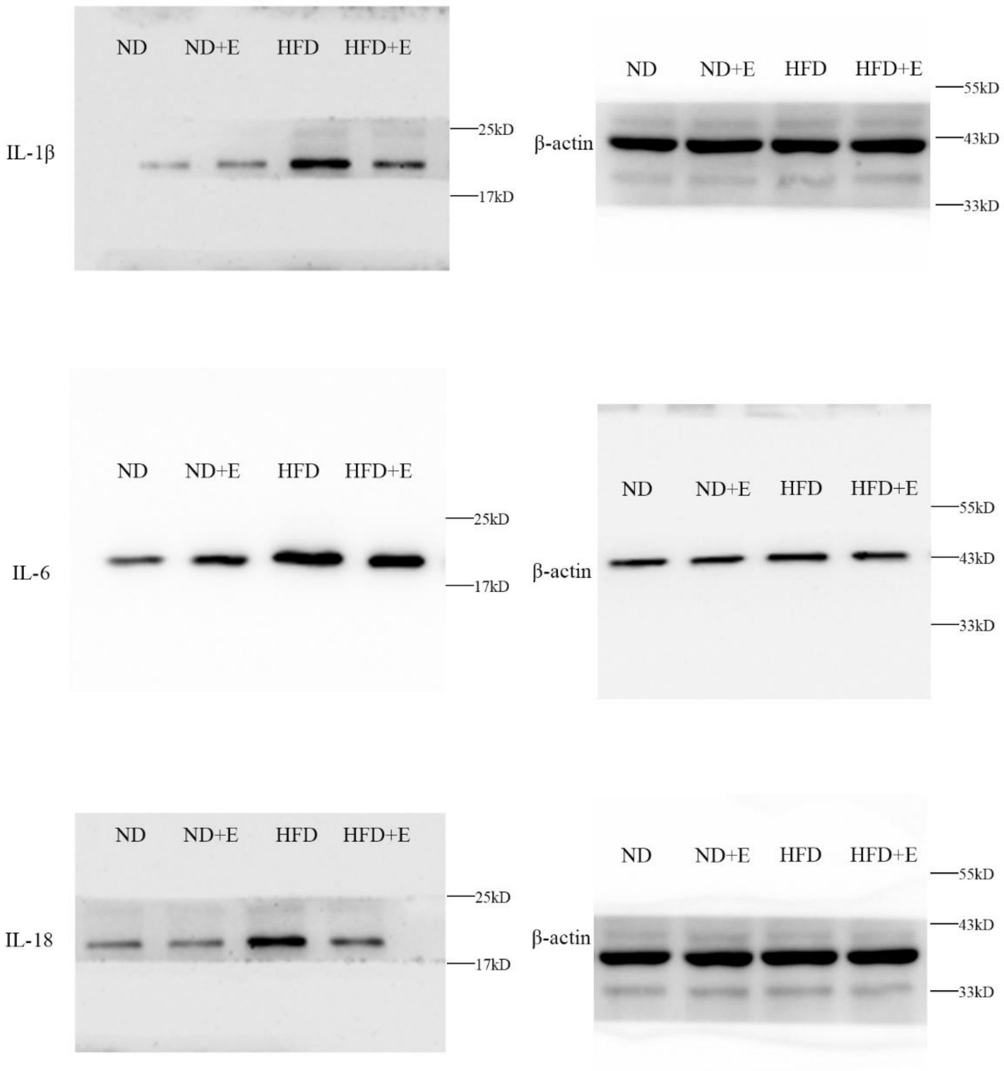

Figure S7. The IL-1β, IL-6, and IL-18 original gels.

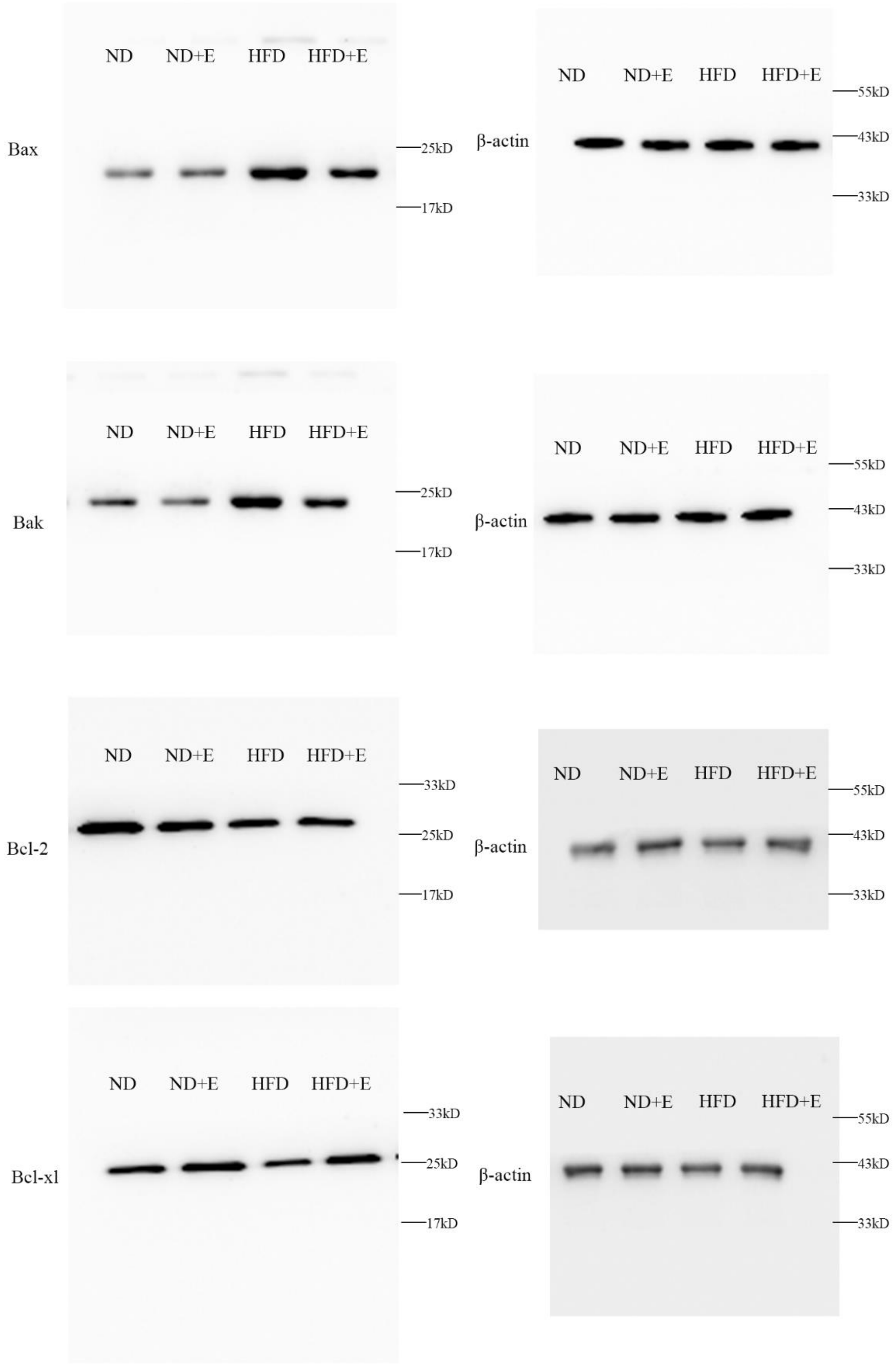

Figure S8. The Bak, Bax, Bcl-2, and Bcl-xl original gels.
